# The Gut–Vagina Axis

**DOI:** 10.3390/microorganisms14061327

**Published:** 2026-06-13

**Authors:** Lorenzo Agoni, Elena Roselletti, Giovanni Marasco, Canio Martinelli, Eva Pericolini, Francesco De Seta

**Affiliations:** 1 Unit of Obstetrics and Gynecology, Fondazione Poliambulanza Istituto Ospedaliero, 25124 Brescia, Italy; lorenzo.agoni@poliambulanza.it; 2 Department of Biosciences, Faculty of Health and Life Sciences, Medical Research Council Centre for Medical Mycology, University of Exeter, Exeter EX4 4QD, UK; 3 Department of Medical and Surgical Sciences, University of Bologna, 40138 Bologna, Italy; 4 Sbarro Institute for Cancer Research and Molecular Medicine and Center of Biotechnology, College of Science and Technology, Temple University, Philadelphia, PA 19122, USA; 5 Unit of Obstetrics and Gynecology, Department of Human Pathology of Adult and Childhood “Gaetano Barresi”, University of Messina, 98124 Messina, Italy; 6 Department of Surgical, Medical, Dental and Morphological Sciences with Interest in Transplant, Oncological and Regenerative Medicine, University of Modena and Reggio Emilia, 41124 Modena, Italy; 7 Department of Obstetrics and Gynecology, IRCCS San Raffaele Scientific Institute, 20132 Milan, Italy

**Keywords:** microbiome, gut–vagina axis, vaginal microbiome, gut microbiome

## Abstract

The gut–vagina axis has emerged as a growing area of interest in female health due to its potential role in mediating physiological processes via interactions between distinct microbiomes, including microbial migration, hormonal and immune regulation, and metabolite exchange. Recent advances in microbiome research suggest bidirectional communication between gut and vaginal communities, with potential effects on microbial composition, immune responses, hormonal balance, and metabolic activity in both sites. In this review, we outline the most promising features of the gut–vaginal relationship, emphasize the significance of their plausible bidirectional communication, and discuss how these interactions may affect local and systemic health.

## 1. Introduction

The advent of next-generation sequencing (NGS) technologies, together with the completion of the initial phase of the Human Microbiome Project, has ushered in a new era of microbiome research. Significant attention and effort have been devoted to understanding the composition of microbiomes at various anatomical sites and the relationships between them.

Numerous seminal studies have investigated the composition of both the gut [1,2,3,4,5] and vaginal [6,7,8,9,10] microbiomes, as well as their correlation to health and disease [11,12,13,14,15,16,17,18,19,20,21,22,23,24,25]. Skin and mucosae harbor distinct microbiomes at each body site. For example, besides the gut and vaginal microbiomes, there are also the skin [26,27], scalp [28], oral cavity [29,30], bladder [31,32,33], and male genital [34] microbiomes. Each microbiome has distinct features and can influence its host organ through microbial metabolism and secreted mediators, thereby contributing to overall health. For example, the gut microbiome digests dietary fiber and produces vitamins (including B-group vitamins and vitamin K), supporting intestinal homeostasis and systemic wellbeing. Gut microbes also release signaling molecules that affect distant organs. For example, they can produce neurotransmitters such as GABA, serotonin, dopamine, and norepinephrine that modulate brain function, giving rise to the concept of the gut–brain axis. The gut–brain axis can be defined as the bidirectional network linking the gut (including its microbiome) and the brain via neural, immune, endocrine, and metabolic pathways. Microbial metabolites, neurotransmitters, and cytokines influence mood, cognition, and stress responses, while brain signals affect gut motility and secretion. Dysregulation is implicated in psychiatric and neurological disorders. Dietary and microbiome-based interventions may be effective in modulating this equilibrium for global wellbeing.

Expanding on this concept, several other axes have been proposed. These include the gut–lung axis, through which the gut microbiota modulates pulmonary immunity and susceptibility to respiratory disease, and the gut–skin axis, in which microbial metabolites and systemic inflammation influence conditions such as eczema and acne. The gut–liver axis has also been described, whereby the portal transfer of gut-derived metabolites and LPS affects liver metabolism and disease. Additional examples include the oral–gut axis, through which oral microbes can translocate to the gut and promote inflammation, and the gut–heart axis, in which gut microbial metabolites and systemic inflammation influence cardiovascular risk, atherosclerosis, and cardiac function. Broader interactions have also been described, including the gut–immune and gut–metabolic axes, through which the microbiome shapes systemic immunity, metabolic health, insulin sensitivity, and obesity. For many of these axes, the evidence is preliminary, and they should be viewed as working hypotheses or conceptual frameworks to guide research and interpretation of biological functions. The gut–vagina axis falls into this category: the connection between these two microbiomes and organs is apparent but remains insufficiently studied and not yet completely proven. This interplay has yet to be formally characterized and defined despite its intuitive plausibility.

These proposed axes describe how gut microbes and their metabolites, immune mediators, neural pathways, and endocrine signals can influence distant organs, and conversely how those organs and host states shape gut ecology. For some axes (gut–brain, gut–liver, gut–immune, and gut–metabolic), bidirectional communication is supported by substantial experimental and clinical evidence. In contrast, others (gut–lung, gut–skin, oral–gut, gut–vagina, and gut–heart) are supported by a growing but still more limited body of data. Although mechanistic links and directionality are plausible and increasingly documented, many reported associations remain correlative and require causal validation [35,36,37,38,39,40,41,42,43,44]. Thus, while bidirectionality is a useful organizing principle, its strength and mechanistic clarity vary by axis: some are well established, while others remain working hypotheses guiding ongoing research.

Conceptually, the gut–vagina axis does not differ from other proposed gut–organ axes, as it implies bidirectional communication between the two organs, potentially mediated by interactions among the microbiome, immune, endocrine, and metabolic pathways. In this review, we summarize current knowledge of the gut and vaginal microbiomes, with particular emphasis on their plausible interrelationship. We propose and discuss a formal definition of the gut–vagina axis to help frame the field’s boundaries and guide research directions, highlight its key features, and review the supporting evidence. Clarifying the mechanisms underlying these interactions will yield valuable insights for developing targeted strategies to prevent and manage diseases in women.

## 2. Definition and Significance

**Figure 1:**
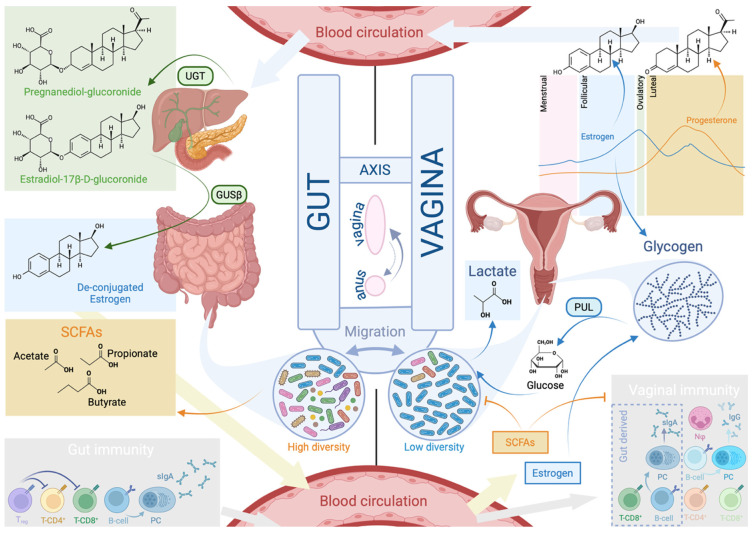
The gut–vagina axis. In the center, the connection constituting the gut–vagina axis is shown with highlights on the migration of bacteria from one microbiome to the other. On the left, the gut side of the axis displays its most relevant features, including a high-diversity microbiome that can migrate to the vaginal microbiome, deconjugate estrogens via β-glucuronidase (GUSβ), and produce short-chain fatty acids (SCFAs). These metabolites may recirculate systemically and reach the vaginal environment, where estrogens, in particular, may exert their action on the vaginal microbiome. The immune cells playing a role in host defense are shown, particularly: neutrophils (Nφ), T cells (T_reg_, T-CD4^+^, T-CD8^+^), B cells, plasma cells (PCs), and secretory IgA antibodies (sIgA). On the right, the vagina side of the axis shows a low diversity microbiome, glycogen metabolized to glucose by pullulanase enzyme (PUL), lactate produced by lactobacilli upon utilization of glucose, and immune cells, including neutrophils (Nφ), T cells of both gut and vaginal origin (T-CD4^+^, T-CD8^+^), B cells of both gut and vaginal origin, and plasma cells (PCs) of both gut and vaginal origin, producing secretive IgA antibodies (sIgA) and IgG antibodies (IgG). Created in https://BioRender.com.

We propose here a definition of the gut–vagina axis as the continuous bidirectional communication between gut and vagina microbiomes that constitutes an interdependent homeostasis of the two systems. (Figure 1). This definition is not a synthesis of the bulk of research data, which are currently scant, but rather a framework and working model, based on preliminary evidence and plausible hypotheses, to organize future studies and obtain the necessary data and confirmation.

We will now list the features that comprise this definition to better characterize them and prepare for the discussion developed throughout the article.

First, the concept of “communication” implies possible crosstalk mechanisms operating at multiple levels, including: (1) microbial migration, (2) regulation of hormone levels, (3) exchange of metabolites, and (4) modulation of the immune response. All these features are presented and discussed in this article.

The interaction between the gut and vaginal microbiomes is theoretically “continuous” and dynamic, shifting with hormonal changes during the menstrual cycle and across life stages. This relationship is driven not only by hormonal fluctuations but also by gut-derived enzymatic activity, as explained below, highlighting the interconnected nature of the two microbiomes.

Communication between the gut and vaginal microbiomes is considered potentially “bidirectional.” While evidence for microbial migration from the gut to the vagina is strong, movement in the opposite direction is less well substantiated. Nevertheless, studies show that the rectum and vagina share many bacterial species, suggesting some exchange between these sites [45,46].

This communication may support an “interdependent homeostasis,” in which the gut and vaginal microbiomes do not function in isolation but rely on each other to maintain dynamically stable local environments. Consequently, alterations in one system could directly or indirectly affect the other, with implications for overall health and function.

A deeper understanding of the interplay between the gut and vaginal microbiomes could enable more effective optimization of their functions than addressing each microbiome separately. By targeting and modulating the gut–vagina axis, it may be possible to improve overall health outcomes and refine strategies for disease prevention and management.

Before reviewing the evidence for the existence of the gut–vagina axis as defined above, we first present the background needed to understand the composition and functions of the gut and vaginal microbiomes, with the aim of framing the axis’s roles.

## 3. The Vaginal Microbiome

The vaginal microbiome is a distinct, active microbial community that plays a central role in women’s reproductive health. In healthy, reproductive-age women, *Lactobacillus* species predominate, comprising the majority of the microbiome [47].

NGS studies classified the vaginal microbiome into five major vaginal community state types (CSTs): CST-I, CST-II, CST-III, and CST-V, which show prevalence of specific lactobacillary species (*L. crispatus*, *L. gasseri*, *L. iners*, and *L. jensenii*, respectively), and CST-IV, which is depleted of lactobacilli and is characterized by a diverse microbiome that may show prevalence of *Gardnerella*, *Fannyhessea*, *Prevotella*, and other species. CST-IV constitutes a condition of vaginal dysbiosis with increased susceptibility to infections [6,48,49,50,51]. The distribution of CSTs differs among various groups and is shaped by factors such as ethnicity, location, and socioeconomic status, which may play a role in the prevalence of infections [48,49,52].

Lactic acid in the vaginal environment lowers the pH to 3.5–4.5, helping prevent proliferation of non-lactobacillary bacteria and viruses [53,54]. Lactic acid has been proven effective, for instance, against HIV [55], HPV [56], and bacterial vaginosis [57]. The presence of lactobacilli (CST-I-II-III-V) is physiological and generally protective compared with their absence (CST-IV) [58].

Lactobacillary activity fluctuates across the ovarian cycle and life stages. Puberty, pregnancy, the postpartum period, and menopause are all associated with profound, hormone-driven microbial shifts [49]. Estrogen levels are important because vaginal epithelial cells produce glycogen in response to estrogen. Adequate estrogen thus ensures glycogen availability for lactobacilli metabolism. Consequently, during the menstrual phase, from the late luteal through the early proliferative phase, estrogen and glycogen levels are low, which can reduce lactobacilli proliferation and function and favor the occurrence of various forms of vaginitis [59,60,61,62]. These physiological fluctuations show that vaginal health is not defined by a single “ideal” microbiome but by the ecosystem’s resilience to environmental and systemic perturbations.

Besides defending the vaginal environment from pathogens, lactobacilli help maintain the integrity of the vaginal mucosa against invasion. An optimal vaginal microbiota, typically CST-I, has been associated with better health outcomes, including lower rates of pre-term labor [20,63] and reduced acquisition of sexually transmitted infections (STIs) such as HIV [64,65], gonorrhea [66,67], trichomonas [68,69], and chlamydia [69,70].

Not all lactobacillus-dominated vaginal microbiomes are equivalent. CST I, dominated by *L. crispatus*, is associated with the most favorable outcomes for protection against pathology and overall reproductive health, whereas CST III, dominated by *L. iners*, is often linked to outcomes closer to dysbiosis, such as higher risk of preterm delivery and reduced protection against STIs. For this reason, it has been questioned whether *L. iners* should be classified as a eubiotic or dysbiotic determinant. Because CST II and CST V—dominated by *L. gasseri* and *L. jensenii*, respectively—are less prevalent than CST I and CST III, their role in vaginal eubiosis is less well established; they are thought to be protective but likely confer less resilience than CST I.

## 4. The Gut Microbiome

### 4.1. The Gut Microbiome

The human gut microbiome is a highly complex ecosystem of trillions of microorganisms, primarily bacteria from the phyla *Firmicutes*, *Bacteroidetes*, *Actinobacteria*, and *Proteobacteria*, as revealed by large-scale initiatives such as the Human Microbiome Project [1]. It plays a vital role in regulating host metabolism, immunity, and endocrine function by interacting with host tissues through diverse metabolic and signaling pathways, thereby influencing overall health and disease susceptibility [71,72].

The gut microbiota helps maintain intestinal health by producing short-chain fatty acids (SCFAs) such as acetate, propionate, and butyrate, which regulate host metabolism and immune responses. Butyrate is especially important as an energy source for coloncytes and for supporting the intestinal barrier. SCFAs activate G-protein-coupled receptors (e.g., GPR41/43) to influence host energy balance and gluconeogenesis, as shown in in vivo studies [73,74]. The gut microbiome profoundly influences host metabolism by modulating energy harvest, nutrient processing, and signaling pathways. In a landmark in vivo study, transplantation of microbiota from obese mice into germ-free recipients transferred the obesity profile, implicating microbial communities in enhanced energy harvest [14]. When gut microbial balance is disrupted (dysbiosis), SCFA production can decrease, the barrier may weaken, and systemic inflammation and metabolic disturbances can increase [75,76]. In vitro and in vivo studies show that butyrate enhances tight-junction protein expression and epithelial barrier function in cultured intestinal epithelial cells [77].

In vivo experiments in mice demonstrate that decreased SCFA availability and dysbiosis compromise barrier integrity and promote translocation of microbial products, driving systemic low-grade inflammation and metabolic disturbances. For example, mouse studies linking high-fat diet-induced dysbiosis to increased plasma lipopolysaccharide (LPS) (metabolic endotoxemia) and insulin resistance provide mechanistic evidence for this pathway [76]. Additional murine work shows butyrate’s role in maintaining barrier homeostasis and regulating immune tolerance [78].

Human studies report associations consistent with these mechanisms but are largely correlative. Metagenomic analyses link altered microbiome composition and reduced SCFA-producing taxa to metabolic syndrome and type 2 diabetes [79], and clinical studies correlate biomarkers of barrier dysfunction and microbial translocation with systemic inflammation in metabolic disease cohorts. More recently, strain-specific gut microbial signatures in type 2 diabetes were identified in a cross-cohort analysis of 8117 metagenomes [80]. This large human metagenomic study reports reproducible alterations in gut microbial composition in type 2 diabetes and documents depletion of several butyrate/SCFA-producing taxa and strain-level functional changes linked to glycometabolic status. Taken together, these in vitro, in vivo, and human data support a pathway from dysbiosis to reduced SCFAs, which leads to barrier impairment and eventually ends in systemic inflammation and metabolic derangement, though causal confirmation in humans remains limited.

The microbiome also shapes immune development and responses through multiple mechanisms. Experimental in vivo work in mice showed that specific commensals can induce regulatory T cells and modulate mucosal immunity [81], and reviews synthesize how microbial molecules instruct immune tolerance and inflammatory set points [82]. Disruption of the gut barrier with translocation of microbial products such as LPS has been demonstrated in animal models to drive systemic low-grade inflammation linked to insulin resistance and metabolic disease [76]. Human observational studies further associate markers of microbial translocation and altered microbiota composition with metabolic inflammation, though causality in humans remains less established [83,84].

Finally, the gut microbiome interacts with endocrine and xenobiotic pathways to affect distant organ physiology. In vivo mouse studies have shown that microbial modification of bile acids alters signaling through nuclear receptors (FXR, TGR5), with downstream effects on host lipid and glucose metabolism [85]. Microbial enzymes can also biotransform drugs and drug metabolites: preclinical work combining in vitro enzyme assays and in vivo mouse models demonstrated bacterial reactivation of an anticancer drug metabolite and that inhibiting the responsible bacterial enzyme reduced host toxicity [86,87]. Human studies increasingly document associations between microbiome composition and endocrine or pharmacologic outcomes, but mechanistic confirmation often relies on complementary in vitro and in vivo experiments.

### 4.2. The Estrobolome

Over the past few decades, research has increasingly shown that the gut microbiome can modify circulating steroid hormone levels by transforming hormones via microbial enzymes. This concept, termed the “estrobolome,” refers to the collection of gut microbes and microbial genes that metabolize estrogens. The estrobolome plays a key role in regulating systemic estrogen levels and may influence the development of estrogen-dependent diseases [88,89,90].

Estrogens play a pivotal role not only in female reproductive physiology but also in diverse systemic processes, including energy metabolism, bone homeostasis, cardiovascular function, aging, and immune regulation. Their pleiotropic effects are mediated by estrogen receptors expressed in multiple tissues, and the net hormonal milieu is determined by synthesis, peripheral conversion, tissue uptake, receptor signaling, and clearance mechanisms [91,92,93,94,95,96]. Perturbations in circulating estrogen levels or in tissue responsiveness can therefore have widespread consequences for health and disease susceptibility.

Following receptor-mediated actions, estrogens are subjected to hepatic phase II metabolism, in which polar groups are enzymatically appended to the steroid nucleus to increase water solubility and facilitate excretion. The principal phase II reactions for estrogens are glucuronidation and sulfation. Glucuronidation is catalyzed by the UDP-glucuronosyltransferase (UGT) family, which transfers glucuronic acid from UDP-glucuronic acid to hydroxyl groups on estradiol and estrone, producing estrogen glucuronides. Sulfation is mediated primarily by cytosolic sulfotransferases (SULTs), which transfer a sulfonate group to form estrogen sulfates.

These conjugated derivatives are actively secreted into bile via hepatic transporters for bile flow excretion. Conjugation thus converts lipophilic parent hormones into forms readily handled by biliary and renal elimination pathways, serving as a major mechanism for estrogen inactivation and clearance from the enterohepatic circulation [97,98].

Within the intestinal lumen, certain gut bacteria express β-glucuronidase (GUS) enzymes that hydrolyze estrogen glucuronides, regenerating free, biologically active estrogens that can be reabsorbed across the intestinal epithelium and re-enter systemic circulation via enterohepatic recirculation [86,88]. Bacterial GUS genes are found in a subset of gut taxa, mainly within the *Firmicutes*, *Bacteroidetes*, and *Proteobacteria* phyla, and encode diverse structural classes with varying substrate specificities that determine their efficiency in deconjugating steroid glucuronides. In addition to β-glucuronidases, some gut bacteria express sulfatases, particularly arylsulfatases, capable of hydrolyzing estrogen sulfates.

The net impact of microbial deconjugation on systemic estrogen exposure depends on multiple interacting factors. Key determinants include the abundance and enzyme expression levels of GUS- and sulfatase-harboring taxa, the structural types of GUS enzymes present (which affect access to and turnover of specific substrates), intestinal transit time (longer transit increases opportunity for deconjugation), and the composition of the bile acid pool, which influences both substrate delivery and microbial community structure. Host variables such as diet (fiber, phytoestrogens), antibiotic use, medication (e.g., UGT inducers/inhibitors), and genetic variation in hepatic conjugating enzymes further modulate the balance between conjugation and microbial deconjugation.

Experimental evidence supporting these pathways comes from complementary approaches. In vitro enzymology and fecal enzyme assays have demonstrated direct deconjugation of estrogen glucuronides by isolated bacterial GUS enzymes and fecal extracts [86]. In vivo animal studies show that manipulating the gut microbiome or inhibiting bacterial GUS activity alters circulating and tissue estrogen levels and modifies physiological outcomes (e.g., drug toxicity, hormone-responsive phenotypes). Human observational and metagenomic studies correlate variation in GUS-harboring taxa and fecal GUS activity with differences in estrogen metabolites and with estrogen-related clinical states, although longitudinal and interventional human data to prove causality remain limited.

Because microbial deconjugation can materially alter the pool of bioavailable estrogens, the composition and functional capacity of the gut microbiome constitute important modulators of systemic estrogen exposure. Changes in microbial diversity or in the prevalence of β-glucuronidase-producing species may therefore influence estrogen-dependent physiological states and disease processes [88,89,90,99].

Notably, the gut microbiome and estrogen metabolism influence each other, and hormonal changes during puberty, pregnancy, and menopause can shift the gut’s microbial balance. This highlights the bidirectional crosstalk between the two microbiomes [100,101].

Research increasingly shows that disruptions of gut microbial balance, particularly changes in diversity and shifts in β-glucuronidase-producing bacteria, can alter estrogen metabolism and affect systemic hormone levels. Such changes have been linked to estrogen-related conditions, including breast cancer, endometriosis, metabolic syndrome, and polycystic ovary syndrome (PCOS). Elevated β-glucuronidase activity can increase estrogen reactivation and raise circulating levels, whereas reduced microbial diversity may lower systemic estrogen. Dysregulation of estrogen metabolism by the gut estrobolome is therefore proposed as a mechanism linking microbial composition to the development and progression of hormone-related diseases [89,99,102,103,104].

The relationship between gut microbes and estrogen metabolism is supported by mechanistic biochemical work showing that bacterial β-glucuronidase enzymes can deconjugate estrogen glucuronides and regenerate active estrogens in the intestinal lumen. Detailed in vitro enzymology and structural studies have characterized dozens of human gut GUS enzymes and demonstrated their ability to convert estrone-3-glucuronide and estradiol-17-glucuronide back to free estrogens, and have shown that this activity can be measured and inhibited in enzyme preparations and fecal extracts.

Conversely, host hormonal state alters gut microbial composition. Human cohort and population studies report predictable shifts in the gut microbiome across life stages when estrogen exposure changes, for example during puberty, pregnancy, and menopause, indicating that sex-hormone milieu is an important driver of microbiome structure and function. Large population analyses have shown menopause-associated changes in gut microbiome composition and functions relevant to hormone metabolism and cardiometabolic risk, supporting the concept that declining estrogen levels, in turn, influence gut ecology.

Clinical observational studies increasingly link disturbed gut microbial communities—notably reduced diversity or relative enrichment/depletion of taxa that harbor GUS genes—to altered systemic estrogen phenotypes and hormone-related disorders. For example, a recent case–control study found higher fecal β-glucuronidase activity in women with polycystic ovary syndrome (PCOS) compared with matched controls and reported correlations between microbial enzyme activities and circulating sex hormones (human, case–control). Such findings are consistent with a model in which changes in gut enzyme activity modify enterohepatic estrogen recycling and thereby influence systemic hormone levels.

Dysregulated gut-driven estrogen levels have been associated with several estrogen-related conditions in humans. Large metagenomic studies of metabolic disease have documented reproducible compositional and functional shifts in the gut microbiome in type 2 diabetes and metabolic syndrome, and cohort and case–control work implicates microbiome alterations in gynecologic conditions such as endometriosis. Although causality is not uniformly established, these human data together with mechanistic in vitro and animal experiments support a pathway linking microbiome composition, microbial enzyme activity (including GUS), altered estrogen availability, and disease risk or progression.

Important caveats and research needs remain. Many human studies are cross-sectional and observational, so reverse causation and confounding (diet, medication, adiposity, age) are concerns; animal and in vitro data provide mechanism but do not always translate (for example, pharmacologic inhibition of certain GUS classes showed biochemical efficacy in vitro but yielded mixed results in tumor models). Future work requires longitudinal human cohorts with paired fecal functional assays, targeted metagenomics/proteomics, and intervention trials (diet, probiotics, enzyme modulators) to establish causality and therapeutic potential. Integrating mechanistic in vitro/in vivo studies with well-controlled human research will be essential to determine whether modulation of gut microbial estrogen metabolism can be exploited to prevent or treat hormone-related diseases.

The gut microbiome can shape the microbial environment of the female reproductive tract by modulating systemic estrogen levels, immune responses, and metabolic signaling. Estrogen is essential for vaginal health, notably by supporting *Lactobacillus* colonization. Changes in gut microbial metabolism that alter estrogen levels may therefore affect the vaginal microbiota, suggesting the gut microbiome acts as a regulator of reproductive-tract microbial balance. Disruptions of the gut microbiota can lead to imbalances and contribute to gynecological disorders.

## 5. Defining the Gut–Vagina Axis: Key Principles and Evidence

**Figure 2:**
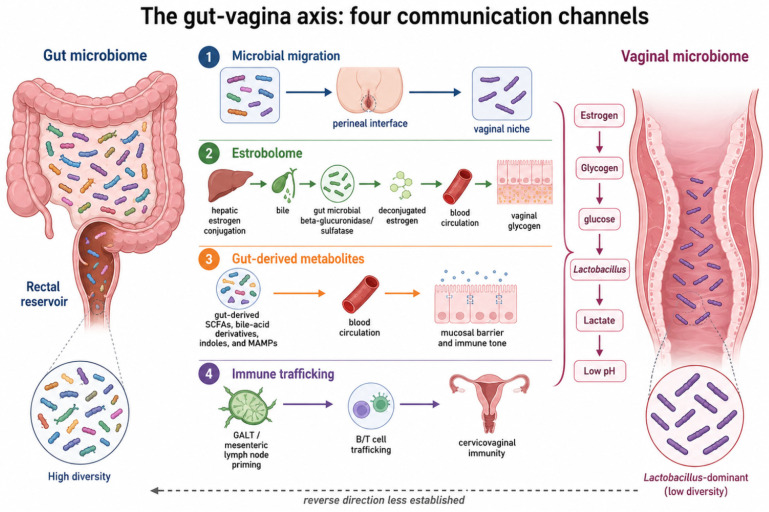
The gut–vagina axis: four communication channels. Schematic representation of the gut–vagina axis as a working model of communication between the gut/rectal reservoir and the vaginal microbiome. Four major communication routes are highlighted: (1) microbial migration, whereby microorganisms from the rectal reservoir may reach the vaginal niche through the perineal interface; (2) estrobolome-mediated endocrine signaling, in which hepatic estrogen conjugates are delivered to the intestine through bile and can be deconjugated by gut microbial β-glucuronidase and sulfatase activities, allowing enterohepatic recirculation and systemic estrogen availability; (3) gut-derived metabolites, including SCFAs, bile acid derivatives, indoles, and MAMPs, which may influence epithelial barrier function and immune tone through systemic host-mediated signaling; and (4) immune trafficking, involving gut-associated lymphoid tissue and mesenteric lymph node priming, followed by B and T cell trafficking toward cervicovaginal immunity. On the vaginal side, estrogen-dependent glycogen deposition supports glucose availability, Lactobacillus dominance, lactate production, and maintenance of a low-pH environment. The figure contrasts the high-diversity gut microbiome with the typically low-diversity, Lactobacillus-dominant vaginal microbiome. Solid arrows indicate better-supported gut-to-vagina pathways, whereas the dashed reverse arrow indicates that vagina-to-gut communication remains less established.

In the sections above, we reviewed the features and functions of the vaginal and gut microbiomes separately. We now reprise the definition of the gut–vagina axis given in the introduction and present the rationale and available evidence underlying each of the four pillars that define the axis: (1) microbial migration, (2) regulation of hormone levels, (3) exchange of metabolites, and (4) modulation of the immune response (Figure 2 and Table 1).

### 5.1. Microbial Migration

**Figure 3:**
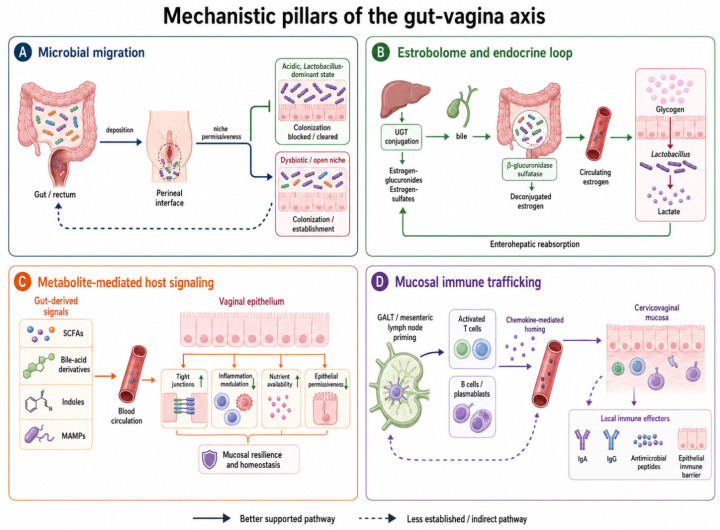
Mechanistic pillars of the gut–vagina axis. Four mechanistic pillars proposed to underlie gut–vagina axis communication. (**A**) Microbial migration. Gut- or rectum-derived microorganisms may be deposited at the perineal interface and subsequently encounter the vaginal niche. Whether these organisms are cleared or establish colonization depends on niche permissiveness: an acidic, Lactobacillus-dominant state favors colonization blockade or transient clearance, whereas dysbiosis or an open ecological niche may permit persistence of gut-derived taxa. (**B**) Estrobolome and endocrine loop. Hepatic conjugation of estrogens, biliary delivery of estrogen-glucuronides and estrogen-sulfates, microbial β-glucuronidase/sulfatase activity, deconjugated estrogen formation, and enterohepatic reabsorption can modulate circulating estrogen. Systemic estrogen availability can then influence vaginal glycogen deposition, Lactobacillus expansion, and lactate production. (**C**) Metabolite-mediated host signaling. Gut-derived SCFAs, bile acid derivatives, indoles, and MAMPs may enter the circulation and influence the vaginal epithelium through effects on tight junctions, inflammation, nutrient availability, epithelial permissiveness, and mucosal resilience. (**D**) Mucosal immune trafficking. Gut-associated lymphoid tissue and mesenteric lymph nodes may prime T cells and B cells/plasmablasts, which can undergo chemokine-mediated homing toward the cervicovaginal mucosa and contribute to local IgA/IgG responses, antimicrobial peptide activity, and epithelial immune barrier function. Solid arrows indicate better-supported gut-to-vagina pathways; dashed arrows indicate indirect, plausible, or less-established feedback routes.

Microorganisms can plausibly migrate from the gut to the vagina due to the close anatomical proximity of the rectum and vaginal introitus, and such translocation can occur throughout a woman’s life (Figure 3A). Mechanical deposition of enteric organisms at the vulvovaginal margin—via toileting, inadequate perineal hygiene, sexual activity, or direct contact—provides the initial inoculum. The probability that these taxa establish likely depends on inoculum size, frequency of exposure, and the local ecological state at the time of contact. Local ecological compliance determines whether deposited organisms persist and expand. The vaginal niche is normally acidic, maintained by glycogen fermentation by dominant *Lactobacillus* species, which inhibits many enteric taxa from settling. When lactobacilli are depleted, for example after antibiotic use or hormonal changes, ecological niches open and permit colonization by a diverse microbiota, which may include enteric bacteria. Microbial traits such as adhesion factors, acid tolerance, oxygen tolerance, glycogen-degrading enzymes, mucin-degrading enzymes, and the ability to utilize vaginal substrates further influence colonization success. Several host-mediated factors may modulate both transfer frequency and niche receptivity. Practices such as douching and hygiene habits, as well as obstetric events, gynecologic procedures, and antibiotic exposure, can disrupt resident communities and mucosal integrity, increasing the likelihood that gut-derived organisms will persist. Hormonal contraception and systemic shifts in estrogen levels may also alter epithelial turnover and glycogen deposition, thereby affecting vaginal microbiota composition. Ecological interactions and community dynamics determine long-term outcomes after initial transfer. Transient detection of gut taxa in the vaginal microbiota often does not equate to stable colonization: resident microbes may competitively exclude newcomers, whereas strains able to integrate into existing metabolic networks can shift community structure and promote dysbiosis. Biofilm formation may facilitate persistence and proliferation in a less permissive environment. Temporal windows of susceptibility include prepuberty, the perimenstrual period, postpartum, and postmenopause.

Evidence for gut-to-vagina migration derives from multiple methodological approaches, beginning with culture-based and targeted PCR studies that documented the same species and, in some cases, identical strains in paired rectal and vaginal samples from the same individuals. Genotyping of isolates from paired vaginal and rectal swabs in pregnant women showed high species-level similarity and strain overlap, supporting the rectum as a reservoir for organisms recovered in the vagina [45]. Early qPCR and culture studies likewise reported strong correspondence in bacterial loads for taxa such as *Lactobacillus* spp., *Gardnerella* and *Atopobium* between the two sites [105]. High-throughput sequencing studies extended these findings by characterizing community composition across body sites and demonstrating taxonomic overlap between rectal and vaginal samples, while high-resolution phylogenetic analyses documented frequent presence of rectal-associated taxa in the vagina during vaginitis or vaginosis states [106,107]. Longitudinal sampling and strain-level metagenomic analyses provide the strongest evidence that rectal populations can precede and seed vaginal populations. A longitudinal, multi-site study of pregnant women showed temporal and spatial variation across maternal body sites, with episodes in which shifts in rectal communities preceded changes in the vaginal microbiome [108]. Strain-level metagenomics applied to mother–infant transmission demonstrated the feasibility of tracking identical species across different maternal body sites, establishing that identical genomic signatures can be traced from gut/rectal reservoirs to other niches [109]. More recently, prospective sampling in late gestation and postpartum reported partial convergence of rectal and vaginal maternal microbiota, with increased sharing of taxa and reduced community distances between sites [110]. An interesting study conducted on male-to-female transsexual women showed that the neovagina became colonized by the same Lactobacillus species found in the rectum, supporting the role of the gut as an important reservoir for vaginal microbiota [111].

Nevertheless, multiple alternative explanations exist for non-lactobacillary vaginal colonization, supported by culture and sequencing studies. One possibility is expansion of resident low-abundance taxa when ecological constraints change: high-resolution sequencing has shown that many taxa associated with bacterial vaginosis (BV) are detectable at low abundance in healthy women and can bloom when community structure shifts, consistent with endogenous overgrowth rather than recent external introduction [106]. Sexual transmission and partner microbiota are implicated as sources of non-lactobacillary organisms. Studies identifying overlap between male genital and female vaginal taxa, and associations between sexual behavior and vaginal community composition, support sexual transfer as a contributor to community change [6]. Similarly, introduction from other local urogenital sites such as urethra or perineal and vulvar skin has been proposed, given documented microbial overlap across adjacent genitourinary niches [108]. Iatrogenic and environmental perturbations offer additional routes to shifts in microbiome composition. Antibiotic exposure, douching, intravaginal products, and obstetric or gynecologic procedures alter local selective pressures and frequently precede shifts toward non-lactobacillary communities. Childbirth and the postpartum period are clear examples: longitudinal studies document marked, sometimes prolonged, changes in vaginal microbiota after delivery, consistent with disturbance-driven community succession [10]. In vitro studies also show that douching can profoundly impair *Lactobacillus* viability [112]. Host factors and ecological interactions can independently favor a non-lactobacillary microbiota. Individual differences in mucosal immunity, mucin composition, epithelial receptor expression, and hormonal levels alter nutrient availability and immune tolerance, enabling overgrowth of opportunistic taxa. Moreover, biofilm formation by species such as *Gardnerella* and associated anaerobes creates resilient communities that resist displacement by lactobacilli and persist after perturbation, a mechanism supported by microscopy and molecular studies demonstrating biofilms in BV [113].

These hypotheses are not mutually exclusive and likely interact: transient bacterial seeding may persist only if host conditions permit, or latent resident taxa may expand following perturbation. Distinguishing among these mechanisms requires longitudinal, strain-resolved studies that integrate behavioral, clinical, and host-immune data to establish directionality and causal drivers.

Vagina-to-gut microbial migration is biologically plausible but the evidence for such a route is limited. Longitudinal studies of maternal body sites during pregnancy report temporal and spatial dynamics with episodes of cross-site similarity, implying possible transfer or shared ecological drivers between vaginal and rectal/gut niches [108]. Strain-resolved metagenomic analyses have demonstrated identical strains across distinct body sites within individuals, confirming that these transfer events can occur [109].

### 5.2. Regulation of Hormone Levels: The Estrobolome

The gut microbiome can influence the vaginal environment primarily by modulating systemic estrogen availability through microbial deconjugation of hepatic estrogen conjugates (Figure 3B). Hepatically conjugated estrogen-glucuronides and estrogen-sulfates are secreted into bile and delivered to the intestine, where bacterial β-glucuronidase and sulfatase activities can hydrolyze them and regenerate free, bioactive estrogens that are available for intestinal reabsorption via enterohepatic recirculation [88,114]. Human cross-sectional and cohort studies have reported correlations between fecal microbiome composition or enzymatic activity and circulating or urinary estrogens and their metabolites, supporting a role for gut microbial activity in shaping systemic estrogen pools [114,115].

Because local estrogen signaling is a major determinant of vaginal physiology, changes in systemic estrogen driven by gut microbial activity can have downstream effects on the vaginal niche. Estrogen stimulates glycogen deposition in vaginal epithelial cells, and glycogen-derived substrates are central to *Lactobacillus* colonization and lactic acid production that maintain a low vaginal pH [116,117]. Observational work shows that lactobacilli-dominant vaginal communities are more frequent when serum unconjugated estrogens are higher, consistent with an endocrine link between systemic estrogen and vaginal bacterial composition [116]. Experimental in vitro studies provide complementary mechanistic evidence of estrogen’s direct effects on vaginal *Lactobacillus* physiology: estradiol exposure modulates membrane dynamics, adhesion to vaginal epithelial cells, and biosurfactant production in *L. crispatus* strains, plausibly enhancing mucosal colonization under estrogenic conditions [117]. Taken together with human epidemiologic data linking gut microbiome features to estrogen metabolite profiles [114,115] and recent reviews synthesizing these findings [118], the evidence supports a pathway whereby gut microbial modulation of estrogen availability can indirectly regulate vaginal glycogen availability, *Lactobacillus* colonization, and thus vaginal community structure and stability.

While these studies establish biologic plausibility and consistent associations, causality in humans remains to be proven. Many human datasets are cross-sectional and subject to confounding by age, adiposity, diet, medication use (e.g., antibiotics or hormone therapy), and enzyme activity is often inferred from taxonomic data rather than directly measured. Future longitudinal, intervention, and paired fecal–vaginal studies with functional assays (e.g., fecal enzyme activity, steroid profiling by mass spectrometry, and strain-level metagenomics) are needed to quantify the magnitude of gut-driven estrogen modulation and its direct impact on vaginal ecology and clinical outcomes.

### 5.3. Exchange of Metabolites

Gut–vagina metabolite exchange is mediated mostly by gut-derived molecules that reach the vaginal mucosa via systemic circulation (Figure 3C). Examples of these include: bile acid derivatives, SCFAs, tryptophan metabolites, and microbial-derived molecules such as lipopolysaccharide (LPS) and other microbe-associated molecular patterns (MAMPs).

Bile acid derivatives, transformed by intestinal microbiota, modulate host signaling pathways, including the nuclear hormone receptor farnesoid X receptor (FXR) and the G protein-coupled bile acid receptor TGR5, and thereby influence systemic metabolic states that can secondarily affect mucosal epithelia and microbial selection at distal sites [85]. *Lactobacillus* species play a critical role in regulating the host metabolism, liver function, and immune health by modulating the FXR and TGR5 pathways. In vivo studies show that *Lactobacillus* species in the gut produce bile salt hydrolase (BSH) enzymes that deconjugate bile acids in the intestine. This enzymatic activity leads to the accumulation of specific secondary bile acids, such as tauro-β-muricholic acid, which act as natural FXR antagonists. By inhibiting intestinal FXR signaling, *Lactobacillus* colonization helps reduce fat accumulation, improve insulin sensitivity, and protect against diet-induced obesity and fatty liver disease [119]. Secondary bile acids such as lithocholic acid and deoxycholic acid act as potent natural activators of the TGR5 receptor on intestinal L cells and immune cells. Once stimulated, the TGR5 pathway triggers the release of Glucagon-like peptide-1 (GLP-1) to improve insulin sensitivity, suppresses pro-inflammatory pathways like NF-κB, and strengthens the gut barrier [120]. Through this intricate biochemical axis, *Lactobacillus* species act as upstream regulators of host metabolism and inflammation. However, a role for these metabolites at the vaginal level has not yet been established.

SCFAs produced in the gut by microbial fermentation of dietary fiber act as systemic signaling molecules through cell-surface G-protein coupled receptors (GPCRs) and epigenetic mechanisms; in vitro and in vivo studies show that SCFAs may alter epithelial cell metabolism and barrier properties, plausibly changing the vaginal niche’s compliance to different taxa [73,121]. A substantial fraction of gut-produced SCFAs enter the mesenteric circulation, achieving systemic distribution to influence peripheral organs. At the cellular level, circulating SCFAs exert physiological effects via two distinct, complementary mechanisms: the activation of specific cell-surface GPCRs and the direct epigenetic modulation of host cells through the inhibition of histone deacetylases (HDACs). In the context of the female reproductive tract, this systemic signaling cascade alters the metabolic phenotype and barrier properties of the vaginal epithelium. While high concentrations of SCFAs within the intestinal lumen are characteristically anti-inflammatory and strengthen tight-junction integrity, their systemic immunomodulatory influence can shift the local permissiveness of the vaginal niche [122]. However, systemic alterations driven by gut dysbiosis or modified circulating SCFA profiles can compromise vaginal epithelial defense mechanisms and alter local nutrient availability, rendering the niche highly permissible to colonizing pathogens. This shift allows strict anaerobes and gut-derived bacteria to displace resident lactobacilli. Once established, these dysbiotic taxa generate excessive local concentrations of vaginal SCFAs. This metabolite accumulation triggers severe epithelial inflammation, compromises mucosal tight junctions, and induces dysbiosis, vaginitis or vaginosis [123,124,125].

Microbially derived tryptophan metabolites, such as indoles and indole derivatives, engage host receptors, specifically the aryl hydrocarbon receptor (AhR), and modulate epithelial function and mucosal homeostasis. Experimental in vivo work links these metabolites to barrier regulation and mucosal responses that could indirectly shape distal mucosal microbiota [126]. Microbially derived tryptophan metabolites generated by gut bacteria enter host circulation to influence vaginal mucosal immunity and reshape the local microbiome. These indole molecules travel through the bloodstream and bind to epithelial AhR within the vaginal tract. AhR activation triggers a protective IL-22 and IL-18 cytokine cascade that reinforces local tight junctions and shields the vaginal mucosa against fungal pathogens like *Candida albicans*. Indeed, in an in vivo study, administration of the microbial metabolite indole-3-aldehyde promoted IL-18 expression and protection against *Candida* infection [127].

Microbe-associated molecular patterns (MAMPs) such as peptidoglycan fragments and lipopolysaccharide (LPS) can cross a compromised intestinal barrier and enter the circulation, where they act as potent signals that reshape host metabolic and barrier responses. Circulating LPS triggers low-grade systemic inflammation, often termed “metabolic endotoxemia”, which has been linked to insulin resistance, adipose inflammation, and altered epithelial function in distal tissues [76]. Translocated peptidoglycan and other MAMPs engage pattern-recognition receptors such as Toll-like receptors and NOD-like receptors on epithelial and stromal cells, modulating tight-junction integrity, mucus production, and epithelial turnover [128,129]. Some authors reinforce and extend these concepts, detailing how barrier dysfunction and MAMP translocation link gut dysbiosis to systemic inflammation and multisystem effects [130,131]. Together, these data indicate that MAMP-driven host responses change the mucosal landscape and substrate availability, so systemic MAMP signaling can secondarily influence vaginal community composition by altering epithelial barrier properties and local compliance.

Together, these classes of metabolites and microbial functions act predominantly via systemic host mediation rather than by bulk direct transfer between luminal compartments; their relative contributions to gut-to-vagina crosstalk remain an active area of research requiring paired metabolic, microbiologic, and clinical studies.

### 5.4. Modulation of the Immune Response: The Gut–Vagina Immune Axis

The vaginal mucosa combines general mucosal defenses with distinctive features that shape local immune responses (Figure 3D). Both innate and adaptive immune cells are present but, unlike the gut mucosa, adaptive responses in the lower female reproductive tract are primed in regional draining lymph nodes rather than in organized mucosa-associated lymphoid tissue such as the gut-associated lymphoid tissues (GALTs). Unlike other mucosal sites, IgG predominates in the vaginal lumen. IgG derives both from serum transudation and local plasma cell production, providing broad opsonizing and neutralizing activity against pathogens [46,132,133,134]. Secretory IgA, while less abundant, performs complementary non-inflammatory functions by coating microbes to limit adhesion and invasion and helping maintain stable colonization by commensal bacteria [133,135]. The relative balance and function of IgG and IgA vary with hormonal state and the menstrual cycle: for example, cervicovaginal IgG levels change across the ovulatory cycle and increase post-ovulation, reflecting endocrine regulation of antibody transudation and local synthesis and influencing susceptibility to infection and microbiota composition [134]. The IgG/IgA ratio may also vary depending on the dominant *Lactobacillus* species [136]. Together, IgG and IgA create a layered humoral environment that both defends against pathogens and fosters a mucosal ecology compatible with beneficial commensals. For example, bacteria associated with BV have been shown to be preferentially coated with IgA [137].

Vaginal epithelial cells produce a range of antimicrobial peptides and chemokines that limit microbial growth and recruit/retain appropriate immune effector cells [135,138]. For example, vaginal epithelial cells secrete antimicrobial peptides (AMPs) such as α- and β-defensins and other molecules that exert direct microbicidal activity against bacteria, fungi, and viruses by disrupting microbial membranes, neutralizing toxins, and interfering with microbial adhesion and biofilm formation [139,140]. These mediators are produced by vaginal epithelial cells in response to hormonal and microbial stimuli, contributing to mucosal barrier function and shaping local microbial communities [132,140]. Experimental in vitro work on human vaginal epithelial cells documents expression and secretion of specific antimicrobial mediators and shows that their levels and activity correlate with resistance to pathogens such as *Candida albicans* and bacterial vaginosis-associated bacteria by disrupting microbial membranes or inhibiting growth, and they also modulate microbial community composition [139,141,142,143,144]. In parallel, epithelial cells produce chemokines such as CCL20 and CCL28 that recruit and retain appropriate immune effectors (e.g., IgA-committed plasmablasts, T cells, and innate lymphoid cells) to the mucosa, shaping local immune surveillance and mucosal antibody localization [135,145,146,147]. These epithelial mediators are hormonally regulated and respond dynamically to microbial and inflammatory cues, thereby linking epithelial antimicrobial action with immune cell trafficking to maintain mucosal homeostasis and defend against pathogens while permitting commensal colonization [132,148].

The gut microbiome is a major educator of the host immune system and can therefore affect distal mucosal immunity, including in the reproductive tract. Studies show that specific gut commensals and microbe-derived molecules shape T cell and B cell differentiation and systemic immune balance [81,82,149]. In vivo work in mice demonstrated that specific commensals direct lymphocyte differentiation: *Bacteroides fragilis* produces polysaccharide A that promotes regulatory T cell development and immune tolerance [81,150], while segmented filamentous bacteria induce mucosal Th17 cells that shape systemic and intestinal inflammatory responses [151,152]. In vitro studies further show that microbial signals modulate T cell and B cell programs: short-chain fatty acids promote colonic regulatory T cells via GPCR and epigenetic pathways [73,153,154]. Gut microbes also instruct humoral immunity. In vivo studies reveal that microbial stimulation drives IgA class switching and mucosal IgA responses via GALT interactions, shaping systemic and mucosal antibody repertoires [155]. These gut-primed B cells and their homing cues can seed distant mucosal sites under appropriate chemokine signals, providing a mechanistic route by which gut immune education influences genital tract antibody landscapes [118,133,135]. Human translational and cohort studies corroborate and extend these findings by linking gut microbiome composition with systemic immune phenotypes and cytokine responses. Population-level analyses associate specific microbial taxa and functional signatures with host inflammatory capacity and circulating cytokine profiles [82,156]. Such gut-driven immune programming occurs in GALT and mesenteric lymph nodes, where antigen presentation induces homing receptors and chemokine-receptor patterns on activated lymphocytes [157].

Crucially, primed lymphocytes are capable of trafficking to non-intestinal mucosal sites, including the genital tract, under defined chemokine/adhesion signals. Experimental adoptive transfer work on animals demonstrated that mesenteric lymph node-derived B cells can localize to the cervix and vagina, and chemokines such as CCL28 are implicated in recruiting IgA-committed plasmablasts and other mucosal lymphocytes to the genital mucosa [135,158]. Primed lymphocytes generated in gut-associated lymphoid tissues can traffic to non-intestinal mucosal sites, including the genital tract, under the control of specific chemokine and adhesion-receptor interactions. Classic work on lymphocyte homing established that imprinting in regional lymphoid tissues determines mucosal homing receptor expression and tissue tropism [159]. In vivo studies showed that lymphocytes activated in mesenteric lymph nodes or Peyer’s patches can migrate and populate distant mucosal surfaces, providing functional linkage between gut immune priming and remote mucosal immunity [123,158,160]. Chemokines play a central role in this trafficking: CCL28 selectively attracts IgA-committed plasmablasts via CCR10 and has been implicated in recruiting these cells to the genital mucosa, thereby seeding local IgA responses with cells originally primed in intestinal sites [135,146]. More recent reviews and experimental work emphasize hormonal regulation of chemokine expression in the female reproductive tract, which can modulate the extent to which gut-primed lymphocytes home to and persist in the cervicovaginal mucosa [132,146].

Hormonal fluctuations driven by ovarian function substantially modulate vaginal immunity. Estrogens tend to enhance humoral and cell-mediated responses in the female reproductive tract—promoting epithelial integrity, antibody transudation, and increased B and T cell activity—whereas progesterone fosters a more tolerogenic, anti-inflammatory milieu that is associated with increased mucosal IgA and a relative Th_2_ polarization, and T_regs_ increase state during certain phases [132,161,162].

The gut microbiome can alter these hormonal signals by modulating systemic estrogen availability through microbial enzymatic activities such as bacterial β-glucuronidases, thereby impacting downstream hormone-dependent immune effects in the vagina [88,114]. Changes in microbial composition or enzyme activity in the gut therefore have the potential to shift estrogen-driven epithelial physiology and associated immune responses at the vaginal mucosa.

Separately, the gut educates systemic mucosal immunity via GALT: microbial antigens and metabolites prime B and T cells in GALT and mesenteric nodes, imprinting homing receptors and functional programs that allow these lymphocytes to traffic to distant mucosal sites, including the genital tract [81,135].

Together, these pathways—hormone modulation by gut microbial enzymes and immune priming in GALT with subsequent lymphocyte homing—connect gut microbes, systemic hormones, and local vaginal immunity, forming a gut–vagina immune axis in which perturbations at either site can reverberate through endocrine and cellular immune networks [81,88,163].

Because the gut microbiome both shapes immune cell differentiation in GALT and influences the patterning of systemic and mucosal immune mediators, perturbations of gut communities can plausibly alter vaginal immunity and thus microbial composition. Reviews and recent syntheses emphasize that sex hormones modulate both epithelial chemokine/antimicrobial expression and lymphocyte trafficking, so gut-driven changes in immune priming may shift the balance between *Lactobacillus*-dominated, protective communities and alternative, dysbiotic states [132,164].

### 5.5. A Continuous Bidirectional Communication and Interdependent Homeostasis

The gut–vagina axis can be viewed as a continuous, bidirectional communication channel in which microbial, hormonal, and behavioral signals flow between anatomically adjacent but functionally distinct mucosal sites, producing an interdependent homeostasis rather than two isolated ecosystems. High-resolution surveys document frequent taxonomic overlap and temporal concordance between rectal/gut and vaginal communities, supporting ongoing exchange and dynamic coupling across time [6,106].

Mechanistic pathways enabling this two-way communication include direct microbial transfer at the perineum (mechanical deposition and strain seeding), systemic transmission of microbially modified molecules (for example, deconjugated steroid metabolites), and host-mediated modulation of niche permissivity via hormonal regulation and epithelial physiology [108,114]. Each pathway both transmits information and changes the recipient environment, enabling feedback loops that reinforce stable states or precipitate shifts.

Evidence for reciprocity includes gut-driven endocrine effects that alter vaginal substrate availability (estrogen-dependent glycogen) and thereby favor or disfavor Lactobacillus dominance, and conversely vaginal events (infection, antibiotic therapy, childbirth) that can alter perineal and gut exposures and community structure [88,108]. Strain-level metagenomic work further shows identical or highly similar genomes shared across rectal and vaginal sites in some individuals, consistent with recent migration and bidirectional exchange [109].

Framing the axis as interdependent homeostasis emphasizes resilience and vulnerability: a robust, lactobacilli-dominated vaginal state coexists with a stable gut community under normal hormonal and behavioral conditions, whereas perturbations (antibiotics, hormonal shifts, diet, or inflammation) can propagate through the axis, altering both local composition and function and increasing disease susceptibility [76,106]. Recognizing this continuous, bidirectional coupling highlights opportunities for interventions that target one site to benefit the other and underscores the need for longitudinal, strain-resolved, and mechanistic human studies to define causality and therapeutic potential.

## 6. Imbalance of the Gut–Vagina Axis

A number of common clinical conditions can perturb either gut or vaginal homeostasis and, in doing so, influence the crosstalk that characterizes the gut–vagina axis (Table 2). In this section, we focus on disorders that alter gut microbiota composition, or systemic hormone metabolism, as well as those that directly affect the vaginal microenvironment, because each of these perturbations can propagate along the gut–vagina axis and ultimately reshape vaginal microbial communities and mucosal health.

Gut disorders such as irritable bowel syndrome (IBS) and gut dysbiosis can destabilize the gut–vagina axis and precipitate vaginal dysbiosis. IBS disproportionately affects women and often presents with female-specific features across reproductive stages with symptoms and extra-intestinal burden that vary with hormonal state [165,166,167,168,169]. Hormonal fluctuations linked to the menstrual cycle and life transitions (e.g., pregnancy, menopause) modulate gut motility, visceral sensitivity, and mucosal physiology, intensifying IBS symptoms during low-estrogen phases and altering gut microbial communities [170,171,172].

The severity and type of IBS symptoms in women are strongly influenced by reproductive life stages and the associated fluctuations in ovarian hormone levels. Symptoms often intensify during the late luteal phase and menstruation when estrogen and progesterone decline. Hormonal changes affect gut motility, visceral sensitivity, and immune responses, contributing to symptom variability. Major reproductive transitions, especially menopause, can further impact IBS, with postmenopausal women reporting more severe symptoms and poorer health-related quality of life [171,172,173,174,175].

Women with IBS often experience gynecological and pelvic symptoms, such as dysmenorrhea, chronic pelvic pain, dyspareunia, and urinary issues. This overlap is explained by pelvic organ cross-sensitization, where inflammation or sensory activation in one pelvic organ can affect pain perception in nearby organs. Research and clinical data indicate that colon irritation can sensitize bladder and reproductive tract pathways, leading to shared symptoms across gastrointestinal and urogenital systems. Consequently, women with IBS tend to seek more gynecological care and undergo pelvic surgeries more frequently than those without IBS [169,176,177,178].

Mechanistically, gut pathology can propagate to the vaginal niche through multiple, interacting routes. Gut dysbiosis can expand rectal reservoirs of non-vaginal taxa and increase the probability of perineal seeding of the vagina; it can also perturb enterohepatic handling of host molecules and shift systemic mediator profiles (including microbial products) that change epithelial permissivity at distant mucosae [88,114,179,180]. Clinical and longitudinal studies document temporal concordance and partial convergence between rectal and vaginal communities in physiologic and disturbed states, indicating the gut as a dynamic source of vaginal colonizers [108,123,179].

Clinically, women with IBS frequently report coexisting gynecological and pelvic symptoms—dysmenorrhea, chronic pelvic pain, dyspareunia, and urinary complaints—reflecting pelvic organ cross-sensitization in which colon irritation alters sensory and inflammatory signaling in adjacent pelvic organs [169,181]. These overlaps increase gynecologic healthcare use and procedures among affected women and suggest that gut disorders can manifest as combined gastrointestinal and urogenital morbidity. Taken together, these observations indicate that a primary gut health problem can disrupt the gut–vagina axis and produce measurable imbalance in the vaginal microbiome.

The vaginal microbiome is maintained in a balanced state (eubiosis) that is essential for vaginal health; deviations from this equilibrium (dysbiosis) can be asymptomatic or manifest as clinical conditions such as vaginitis or vaginosis [182,183]. Disturbances in gut homeostasis can impact vaginal ecology because of perineal proximity and systemic effects of microbial products and host mediators [106,107,123].

Aerobic vaginitis (AV) is characterized by the presence of gut-derived aerobic bacteria in the vagina, often accompanied by reduced lactobacilli, a neutrophilic inflammatory response, and a yellowish, foul discharge. First characterized as a distinct clinical entity to distinguish it from bacterial vaginosis, AV is associated with marked local inflammation and epithelial disruption that can increase susceptibility to secondary infections and symptomatic disease [184,185]. AV is more common after menopause, when estrogen levels decline, but it can also occur in premenopausal women following antibiotic exposure, hormonal changes, or other perturbations of the local ecosystem; AV is associated with increased risks of urinary tract infections and adverse pregnancy outcomes [184,185,186,187,188,189]. The inflammatory milieu and epithelial damage that characterize AV likely underlie these associations by compromising mucosal barriers and promoting pathogen translocation [107,179,182,189,190].

This condition highlights the close connection between the gut and vaginal microbiomes: the bacteria that characterize it are typically gut-derived. Common causative species include Gram-negative taxa such as *Escherichia coli*, *Klebsiella pneumoniae*, and *Proteus mirabilis*, and Gram-positive taxa such as *Enterococcus faecalis*, *Enterococcus faecium*, *Staphylococcus* spp., and *Streptococcus* spp. [107,179].

Management implications follow from this etiology: because AV involves inflammation and often mixed aerobic pathogens rather than the anaerobic overgrowth seen in classic bacterial vaginosis, treatment strategies differ and may require targeted antimicrobial therapy along with measures to restore lactobacillary dominance and mucosal integrity. Recognition of the gut origin of many AV agents highlights the importance of addressing rectal reservoirs, hygiene practices, and any underlying gut dysbiosis when preventing recurrence.

Bacterial vaginosis (BV) is defined by depletion of lactobacilli and overgrowth of a polymicrobial anaerobic community, characteristically including *Gardnerella vaginalis*, *Atopobium vaginae*, *Prevotella bivia*, *Mobiluncus* spp. and others, leading to a thin, grayish discharge with a fishy odor and a shift in vaginal pH and metabolic milieu [191]. BV is clinically important because it is associated with increased risk of adverse outcomes, including preterm birth and enhanced susceptibility to sexually transmitted infections [113,192,193].

In vitro studies indicate that BV often involves adherent polymicrobial biofilms in the vaginal epithelium, frequently with *Gardnerella* as a scaffold, that promote persistence, resistance to clearance, and recurrence [113]. Longitudinal and strain-level analyses show that BV is a dynamic state with frequent transitions and context-dependent stability, influenced by host hormones, sexual behavior, hygiene, and local ecological interactions [8].

The hypothesis that the gut acts as a reservoir for BV-associated organisms is supported by studies finding rectal carriage of BV taxa, including *Gardnerella* and other anaerobes, and by observations of taxonomic overlap between rectal and vaginal samples [106,194]. Metagenomics have, in some settings, identified identical genomes across rectal–vaginal pairs, consistent with episodic transfer [109]. However, important caveats temper a simple gut-to-vagina causation: many BV taxa are also common in the vaginal niche at low abundance and can bloom in situ when ecological controls fail, and shared exposures (e.g., sexual intercourse, hygiene) can produce concordant communities without direct migration. Conversely, these dynamics also imply that vaginal taxa may occasionally migrate to and transiently colonize the gut, consistent with bidirectional exchange under permissive conditions [8,106].

In sum, while the gut may serve as a source or reservoir for BV-associated organisms under permissive conditions, current evidence suggests BV more commonly arises from local ecological disturbance, biofilm formation, and host–environment interactions; the directionality of these anaerobic taxa remains difficult to define [108].

Vulvovaginal candidiasis (VVC), most commonly caused by *Candida albicans* though non-albicans species are increasingly encountered, is characterized by a pronounced and often persistent inflammatory response. Clinical and experimental studies indicate that despite intense neutrophil influx, the acidic, lactic-acid-rich vaginal environment can paradoxically impair effective neutrophil antifungal activity, contributing to symptomatic disease and recurrent episodes [51,195,196,197,198,199,200]. Mucosal cytokine responses and ineffective neutrophil clearance are features of VVC pathogenesis and help explain why inflammation, rather than fungal burden alone, often determines symptom severity [201].

The gut can act as an important reservoir for *Candida* species, including *Candida albicans*, with gastrointestinal colonization documented in humans and shown to seed extra-intestinal niches in animal models. Surveys of the human mycobiome report frequent *Candida* carriage in the gastrointestinal tract and oral cavity, and molecular typing has identified strains shared across body sites, supporting the gut as a potential endogenous source for vulvovaginal colonization [202,203]. In murine in vivo models, intestinal *Candida* colonization predisposes to mucosal and systemic candidiasis under permissive conditions, demonstrating biologic plausibility for gut-to-vagina transmission [204,205].

Clinical and molecular studies link intestinal *Candida* carriage with recurrent or complicated VVC in humans. Case reports and observational cohorts have found concurrent intestinal and vaginal colonization with identical *Candida* strains, and eradication or suppression of intestinal reservoirs has been associated with reduced recurrence in some clinical series, suggesting that gut reservoirs may sustain vaginal reinfection [195,200,206].

The interplay between gut reservoir dynamics and vaginal immunopathology has clinical implications. Antibiotic use, dietary factors, and other perturbations that favor intestinal *Candida* overgrowth can increase the pool of organisms available for perineal transfer, while host factors that impair neutrophil fungicidal function or alter epithelial defenses predispose to symptomatic VVC and recurrence [200,201]. Therapeutically, addressing both local vaginal infection and potential intestinal reservoirs, alongside measures to restore mucosal immunity and ecological resilience, may be required to reduce recurrence and antimicrobial resistance, though controlled trials of integrated gut-and-vaginal strategies are sparse and needed to establish best practice.

Mixed vaginal infections illustrate the complex, bidirectional interactions between gut and vaginal microbiomes and complicate clinical management. Clinical and microbiological studies report frequent co-occurrence of VVC, BV, and AV, with roughly one-third of VVC cases involving bacterial co-infections, which is associated with more severe symptoms and treatment failure [207,208,209,210]. Biofilm formation by BV organisms both promotes persistence of anaerobic bacteria and creates a niche that facilitates *Candida* adherence and mixed-species resilience [113,211,212].

Therapeutically, these interactions matter because standard antifungal agents do not correct underlying bacterial dysbiosis, while broad-spectrum antibiotics used for BV can reduce lactobacilli and promote *Candida* overgrowth and recurrence [206]. Metabolomic and sequencing studies show reciprocal metabolic and ecological interactions, such as changes in local pH, nutrient availability, and antimicrobial compound production, that favor coexistence or sequential blooms of bacteria and fungi [8,213].

Finally, the gut can act as a reservoir that sustains repeated perineal seeding of both bacterial and fungal agents, linking intestinal dysbiosis to recurrent mixed vaginal infections; this adds another layer of complexity and suggests that durable management may require addressing gut reservoirs and restoring ecological resilience in both compartments [108].

Current treatment options for recurrent and mixed infections are limited. Although azole-based therapies are usually effective for acute VVC, over half of patients experience recurrences after stopping maintenance therapy. The rise in azole-resistant *Candida albicans* and non-albicans species further complicates management [51,200,208,214,215,216].

## 7. Modulation of the Gut–Vagina Axis

### 7.1. Prebiotics, Probiotics, and Postbiotics

The finding that oral administration of probiotics could benefit the urogenital tract, including BV, AV, VVC, and urinary tract infections (UTIs), provided proof of concept for the existence of a gut–urogenital axis [217]. Common probiotic strains administered in clinical settings include *Lactobacillus reuteri*, *Lactobacillus rhamnosus*, *Lactobacillus crispatus*, *Lactobacillus gasseri*, *Lactobacillus plantarum*, *Lactobacillus acidophilus*, and *Lactobacillus delbrueckii*. These probiotics can be taken orally or applied vaginally in capsule or suppository form. Both deliveries may be effective, but results depend on the strain used, dosage, frequency, route of administration and individual patient differences (Table 3) [218].

Both oral and intravaginal delivery routes have been tested and can be effective in specific settings, but evidence is strain- and product-specific. Oral administration of *Lactobacillus rhamnosus* GR-1 together with *Lactobacillus reuteri* RC-14 was shown to be effective in modifying the vaginal microbiome and augmenting BV treatment along with antibiotics or reducing UTIs in some clinical trials [217,219,220,221,222,223,224]. By contrast, intravaginal administration of *Lactobacillus crispatus* CTV-05 increased the effectiveness of metronidazole therapy and reduced BV recurrence in some multicenter randomized clinical trials [225,226,227,228]. Similar results have been shown for these probiotics for both VVC and vaginal colonization by *Streptococcus agalactiae*, although the evidence is more limited [229,230,231].

Prebiotics such as inulin, lactulose, and pectin serve as nutritional substrates that selectively stimulate the growth of beneficial *Lactobacillus* spp. while inhibiting pathogens like *Candida albicans* [232]. Postbiotics, consisting of non-viable microbes or their components like exopolysaccharides and extracellular vesicles, provide health benefits by enhancing epithelial adherence and creating barriers against pathogen colonization without the risks associated with live bacteria [233,234]. Additionally, synbiotics, which are the combination of prebiotics and probiotics, leverage the synergy between probiotics and prebiotics to enhance the survival and diversity of vaginal lactobacilli [235].

### 7.2. Diet

Healthy gut and vaginal microbiomes may also be supported by diet (Table 3). Reviews have looked into how various eating habits, including vitamin and mineral intake, consumption of fruits and vegetables, dairy products and probiotics, carbohydrates and sugar, grains and fiber, and protein, affect vaginal health. Insufficient intake of vitamins A, C, E, and D has been linked to a higher risk of BV due to these vitamins’ roles in supporting immunity, antioxidants, and the integrity of vaginal tissue [236]. Higher consumption of fruits and vegetables, which supply essential vitamins and plant compounds like betaine, is associated with a lower risk of BV. On the other hand, diets with a high glycemic index may increase BV risk by promoting oxidative stress. Limiting alcohol and animal protein intake, alongside increasing consumption of linolenic acid, can positively influence the vaginal environment by supporting a microbiota predominantly composed of beneficial *Lactobacillus* species [237].

## 8. Discussion

### 8.1. Evidence for a Gut–Vagina Axis

Drawing on the evidence presented in this article and the collective experience of researchers who have advanced the field, the existence of a gut–vagina axis is plausible, though still speculative. Although many researchers in the 20th century isolated fecal bacteria, particularly *Escherichia coli*, from the vagina and urine of both symptomatic and asymptomatic patients, these discoveries were typically interpreted as infections, often attributed to poor personal hygiene, rather than as evidence supporting bacterial migration and communication between neighboring anatomical sites.

Vaginal infections are influenced by interactions between gut microbial reservoirs, host immunity, and hormones. The close proximity of the anus and vaginal opening enables microorganisms, including *Escherichia coli*, BV-associated bacteria, and *Candida*, to transfer from the gut to the vagina, making the gut a key source for both beneficial and harmful microbes [238,239,240,241]. Importantly, these observations also highlight the multikingdom nature of the gut–vagina axis, which involves not only bacteria but also fungi. This broader ecological view may help explain the persistence and recurrence of vaginal infections despite appropriate local treatment.

The observation that orally administered probiotics can improve urogenital outcomes of BV, AV, VVC, and UTIs helped establish the concept of a gut–vagina or, for extension, gut–urogenital axis, whereby microbes or microbe-derived signals translocate or exert systemic effects that alter vaginal ecology and host defenses [217].

Nevertheless, clinical outcomes are heterogeneous. Systematic reviews and meta-analyses report mixed effects overall, while also emphasizing that efficacy depends on strain identity, dose, formulation, treatment duration and timing, and host factors (baseline microbiota, menopausal status, sexual activity, menses, etc.). Consequently, positive results for one strain or formulation should not be generalized to others; higher-quality, strain-specific randomized controlled trials are recommended [242,243,244,245].

With the advent of NGS technology in the early 21st century and the growing recognition of the microbiome concept, researchers began to view microorganisms not merely as potential pathogens, but within the broader context of commensals and pathobionts.

Regarding the vaginal microbiome, seminal work by Jacques Ravel established the typical composition, categorizing it into four community state types (CST-I, -II, -III, and -V) characterized by a predominance of lactobacilli, and one group (CST-IV) marked by a lack of lactobacilli and a highly diverse bacterial community [6]. Many of the bacteria that characterize CST-IV are of gut origin, including *Prevotella*, *Streptococcus*, *Staphylococcus*, *Enterococcus*, and *Bifidobacterium* [7]. This further highlights the biological connection, anatomically facilitated, between the gut and vaginal microbiomes. These bacteria may produce SCFAs, which negatively affect the integrity of the vaginal epithelium and vaginal metabolic homeostasis, and are correlated with unfavorable reproductive outcomes [123]. Additionally, the plasticity of the vaginal system and the variations in bacterial composition within the microbiome suggest a dynamic equilibrium rather than a static state [186,246].

Regarding the gut, the discovery that the gut microbiome can effectively regulate circulating estrogen levels through the expression and activity of β-glucuronidase has revealed a novel systemic role for the gut microbiome [247]. This regulation has a direct impact on the vaginal microbiome, which depends on estrogen for glycogen production to support lactobacilli. Furthermore, imbalanced estrogen production can negatively impact women’s health. Increasing evidence suggests that β-glucuronidase activity may contribute to gynecological conditions such as PCOS and endometriosis [102,103,104] or even cancer, particularly of the breast, endometrium, ovary, and cervix [248].

Finally, the precise regulation of the unique mucosal immunity in the vagina is influenced by the activity of the gut microbiome, particularly through circulating estrogen levels, to which β-glucuronidase contributes, as well as by the recirculation and homing of adaptive T cells and B cells primed in the GALT, all of which support vaginal immunity.

All the discoveries mentioned above underscore the existence of communication between the gut and vagina, and the interaction between these two microbiomes confirms the presence of a gut–vagina axis. Despite growing research, there is still no widely accepted definition of the “gut–vagina axis.” Recently, several efforts have been made to clarify and define the nature of this axis. For instance, Ravel, in a 2016 editorial, briefly mentions the mere existence of a “gut–vagina axis” as an example of the interactions between microbiomes of various body sites [249]. In 2024, Ashonibare refers to a “gut microbiota-gonadal axis” as a form of crosstalk between microbiomes but does not provide a definition [250]. Takada frequently references the concept of a “vagina–gut axis” or, more broadly, “female reproductive tract–organ axes,” which encompass ideas such as the “vagina–gut axis,” “uterus–gut axis,” “ovary–gut axis,” and “vagina–bladder axis.” The authors briefly discussed the translocation of microorganisms between these organs and other components of the axis, including the estrobolome and immune regulation, but elude to provide a clear definition [46,133]. Recent reviews further frame this as a component of broader gut–organ crosstalk and mucosal immune interactions [251].

Broad body-site surveys and high-resolution vaginal microbiome studies show that while gut and vaginal communities are generally distinct, overlap and transient sharing of taxa occur, particularly following perturbations such as antibiotics, sexual activity, childbirth, or other disruptions [8,252]. Targeted sequencing work has identified rectal-associated taxa in vaginal samples during dysbiotic states, suggesting movement across the perineal interface under permissive ecological conditions [106,107]. Current data support episodic transfer and short-term colonization under permissive conditions, but more strain-resolved, longitudinal studies integrating behavioral and host factors are needed to determine whether the vagina is a significant, sustained source of gut microbes in adult women.

In light of all this evidence and reasoned speculation, we propose here a definition of the gut–vagina axis as the continuous bidirectional communication between gut and vagina microbiomes that constitutes an interdependent homeostasis of the two systems. This crosstalk involves microbial migration, hormone regulation, metabolite exchange, and immune system modulation. The relationship is dynamic, changing with hormone levels and influenced by gut activity. Evidence supports microorganism movement mainly from the gut to the vagina, with some shared bacterial species between the vagina and rectum. In fact, the origin of the vaginal microbiome from the gut after birth further proves the connection of gut and vaginal microbiomes [253]. Changes in one microbiome impact the other, maintaining interdependent homeostasis for optimal health.

**Figure 4:**
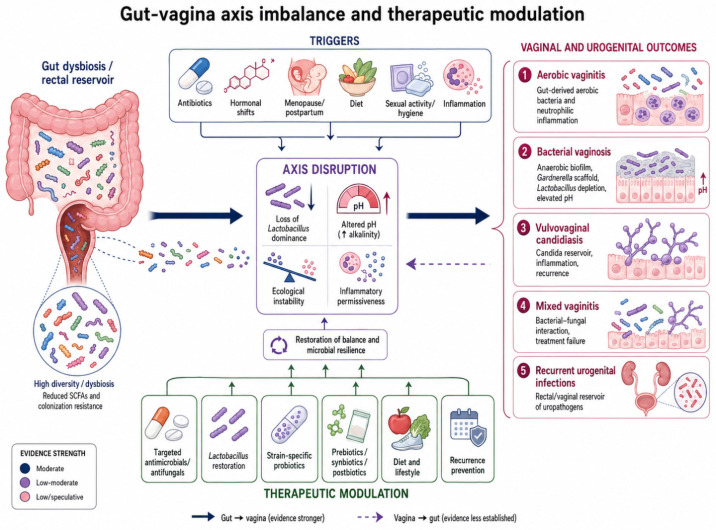
Gut–vagina axis imbalance and therapeutic modulation. Clinical translation of gut–vagina axis imbalance and potential therapeutic modulation. Perturbing factors, including antibiotics, hormonal shifts, menopause/postpartum transitions, diet, sexual activity or hygiene-related exposures, and inflammation, may disrupt gut and vaginal microbial homeostasis. Gut dysbiosis and the rectal reservoir may contribute to axis disruption through loss of Lactobacillus dominance, increased vaginal pH, ecological instability, reduced colonization resistance, and inflammatory permissiveness. These changes may intersect with major vaginal and urogenital phenotypes, including aerobic vaginitis, characterized by gut-derived aerobic bacteria and neutrophilic inflammation; bacterial vaginosis, characterized by anaerobic biofilm formation, Gardnerella-associated scaffolding, Lactobacillus depletion, and elevated pH; vulvovaginal candidiasis, involving Candida reservoirs, inflammation, and recurrence; mixed vaginitis, involving bacterial–fungal interactions and treatment failure; and recurrent urogenital infections, sustained in part by rectal and vaginal reservoirs of uropathogens. Therapeutic modulation may require layered strategies, including targeted antimicrobials or antifungals, restoration of Lactobacillus dominance, strain-specific probiotics, prebiotics/synbiotics/postbiotics, diet and lifestyle interventions, and recurrence-prevention strategies. The evidence-strength legend summarizes the relative maturity of evidence across mechanisms and clinical applications. The solid gut-to-vagina arrow indicates that this direction is better supported, whereas the dashed vagina-to-gut arrow indicates less-established reverse communication.

Taken together, these findings support a model in which vaginal infections arise from the convergence of local vaginal factors and distal gut-derived microbial and immunological influences. Recognizing the gut as a reservoir and regulator of vaginal microbial homeostasis has important implications for the prevention and management of recurrent vaginal infections and underscores the need for therapeutic strategies that address the gut–vagina axis as an integrated biological system (Figure 4).

### 8.2. Key Gaps and Research Priorities

Despite this convergent body of evidence, important caveats remain (Table 4). Many sequencing studies are observational and cannot definitively prove directionality; detection of the same species at two sites can reflect shared exposures rather than recent transfer. Culture-independent strain-level approaches mitigate this limitation by demonstrating near-identical genomes in paired samples, but such analyses are still relatively few and often limited by sampling depth and potential contamination. Moreover, not all gut microbes are fit to colonize the vaginal environment, and successful establishment depends on compatibility with vaginal physicochemical conditions and resident microbiota. Evidence for consistent, clinically relevant gut-to-vagina seeding is stronger for some taxa than others, and many human studies are observational and subject to confounding such as shared exposures and sampling contamination. Controlled longitudinal studies with strain-level resolution and careful sampling protocols are needed to quantify migration frequency, identify high-risk behaviors or states, and determine the clinical significance of gut-derived vaginal colonization.

Despite ongoing research, key scientific and clinical challenges remain in fully understanding the mechanisms of the gut–vagina axis and implementing related therapeutic solutions. Questions persist about the extent and impact of microorganism migration between the gut and vagina, particularly from the vagina to the gut, as well as the roles of immune cell trafficking, β-glucuronidase activity, and SCFA secretion in regulating vaginal health.

Therapeutic approaches such as probiotics face significant challenges in establishing both safety and efficacy, including inconsistent colonization, high recurrence rates, and the lack of robust evaluation frameworks—particularly in vulnerable populations such as pregnant women, immunocompromised individuals, and newborns. Diverse, well-designed clinical trials and deeper genetic and metabolic characterization of candidate species are needed to advance the field and establish reliable interventions.

## 9. Conclusions

Recent studies highlight a bidirectional gut–vagina axis—continuous communication between the gut and vaginal microbiomes that maintains interdependent homeostasis—mediated by microbial migration, hormone regulation, metabolite exchange, and immune modulation, with strongest evidence for microorganism movement from the gut to the vagina; disruptions of this axis are associated with adverse outcomes, including impaired fertility and increased risk of gynecologic cancers. Key challenges remain, including quantifying microbial migration, defining immune cell roles, and optimizing probiotic interventions, and progress will require more diverse clinical trials and deeper microbial characterization. Recognizing the gut as a reservoir for vaginal health underscores the need for integrated approaches to manage and prevent recurrent vaginal infections and for intensified research into the axis’s underlying mechanisms.

## Figures and Tables

**Table 1:** Evidence map of the proposed gut–vagina axis.

Axis Component	Main Mechanism	Best Supporting Evidence to Emphasize	Directionality	Evidence Strength	Clinical Relevance	Main Limitation/Reviewer-Sensitive Point
Microbial migration	Rectal/perineal seeding of vaginal niche; transient deposition may become stable colonization when the vaginal ecosystem is permissive.	Paired rectal-vaginal studies, qPCR/culture overlap, longitudinal multi-site studies, and strain-level analyses showing shared or near-identical taxa across body sites.	Mostly gut/rectum -> vagina. Reverse vagina -> gut remains plausible but much less substantiated.	Moderate for gut -> vagina; low for reverse direction.	AV, BV-associated taxa, recurrent VVC, recurrent UTI/urogenital infections, mixed vaginitis.	Species overlap can reflect shared exposure, sexual transfer, contamination, or local expansion of resident low-abundance taxa; strain-resolved longitudinal data are still limited.
Estrobolome/hormone regulation	Gut microbial beta-glucuronidase and sulfatase deconjugate estrogen metabolites, enabling enterohepatic recirculation and modulation of systemic estrogen availability.	Mechanistic enzymology, fecal enzyme/metagenomic studies, human associations between fecal microbiome and estrogen metabolites, and data linking estrogen to glycogen, Lactobacillus dominance, and lactate production.	Gut -> systemic estrogen -> vaginal niche; host hormone state also feeds back on gut microbiome composition.	Moderate.	Menstrual-cycle shifts, menopause, pregnancy/postpartum transitions, Lactobacillus resilience, pH stability, hormone-related gynecologic phenotypes.	Human causality is incomplete; many studies infer function from taxa rather than measuring enzyme activity and steroid profiles directly.
Metabolite exchange	Gut-derived SCFAs, bile acid derivatives, tryptophan metabolites/indoles, and MAMPs enter circulation and alter epithelial barrier function, immune tone, and niche permissiveness.	Strong general gut–systemic evidence; selected reproductive-tract data for SCFAs and AhR/indole pathways; local vaginal SCFA accumulation associated with dysbiosis and inflammation.	Mostly gut -> systemic host signaling -> vagina. Local vaginal metabolite production is important but not necessarily gut-derived.	Low-moderate.	Barrier integrity, inflammation, Candida defense, dysbiosis permissiveness, mucosal resilience.	Direct proof that gut-derived metabolites reach and reshape the human vaginal niche at clinically relevant levels is still limited.
Immune modulation	Gut microbiome educates T and B cells in GALT/mesenteric lymph nodes; primed lymphocytes and immune mediators can influence cervicovaginal immunity.	Animal and translational work on GALT priming, lymphocyte homing, CCL28/CCR10 recruitment, IgA plasmablast trafficking, and hormonal regulation of genital tract immunity.	Mostly gut -> systemic/mucosal immune programming -> vagina; feedback via infection, inflammation, antibiotics, and hormones is plausible.	Low-moderate.	Mucosal defense, IgA/IgG balance, antimicrobial peptides, STI susceptibility, recurrence of infections.	Much of the mechanistic evidence is extrapolated from general mucosal immunology or animal models; paired human gut–vaginal immune datasets are needed.
Interdependent homeostasis	Gut and vaginal ecosystems may form a coupled system in which perturbation of one site alters the other through microbial, endocrine, metabolic, and immune routes.	Taxonomic overlap, temporal concordance, shared perturbation responses, probiotic proof-of-concept, and clinical recurrence patterns.	Conceptually bidirectional, but evidence is asymmetric: gut-to-vagina is stronger.	Low as a full causal model; useful as a working framework.	Integrated prevention/management of recurrent vaginal infections and broader reproductive-health research.	Should be framed as a working model, not as a fully proven causal axis.

**Table 2:** Clinical phenotypes linked to gut–vagina axis imbalance.

Clinical Condition/Phenotype	Typical Vaginal Phenotype	Gut/Axis Connection	Mechanistic Interpretation	Therapeutic Implication	Evidence Caveat
IBS/gut dysbiosis	No single vaginal phenotype; may coexist with pelvic pain, urinary symptoms, dyspareunia, dysmenorrhea, and recurrent dysbiosis.	Gut dysbiosis may expand rectal reservoirs, alter barrier function, change systemic mediators, and interact with reproductive hormone fluctuations.	Gut disorder can influence pelvic organs through microbial reservoirs, systemic inflammation/metabolites, and pelvic organ cross-sensitization.	In recurrent vaginal dysbiosis, consider GI symptoms, antibiotic history, diet, bowel habits, and pelvic comorbidity as part of phenotyping.	Association and plausibility are stronger than direct proof; avoid presenting IBS as a proven cause of vaginal dysbiosis.
Aerobic vaginitis (AV)	Reduced lactobacilli, aerobic/facultative bacteria, inflammation, epithelial disruption, neutrophils, yellowish or foul discharge.	Often involves gut-derived or rectal-reservoir taxa such as Escherichia coli, Klebsiella, Proteus, Enterococcus, Streptococcus, and Staphylococcus.	Perineal seeding plus permissive vaginal ecology after low estrogen, antibiotics, menopause, postpartum change, or epithelial injury.	Differentiate AV from BV; consider targeted antimicrobials plus restoration of lactobacilli and mucosal integrity; address recurrence reservoirs.	Gut origin is plausible and often likely, but stable colonization versus repeated transient seeding may be hard to distinguish.
Bacterial vaginosis (BV)	Lactobacillus depletion, polymicrobial anaerobic overgrowth, Gardnerella-associated biofilm, elevated pH, fishy odor/discharge.	Rectal carriage and rectal-vaginal overlap of BV-associated taxa are reported; gut may act as a reservoir under permissive conditions.	BV often reflects local ecological disturbance and biofilm persistence, with possible contribution from gut/rectal seeding.	Biofilm-aware management; after standard therapy, consider Lactobacillus restoration or strain-specific probiotic strategies to reduce recurrence.	Directionality is uncertain; BV taxa may bloom locally from low abundance without recent gut migration.
Vulvovaginal candidiasis (VVC)	Candida colonization/infection with inflammation, pruritus, discharge; symptoms often driven by host inflammatory response.	Gut can be a Candida reservoir; shared intestinal and vaginal strains have been reported in recurrent/complicated cases.	Intestinal carriage may support repeated perineal transfer, while vaginal immune and epithelial conditions determine symptoms.	For recurrent VVC, consider intestinal reservoir, prior antibiotics, mixed infection, and ecological restoration alongside antifungal therapy.	Controlled trials of integrated gut-and-vaginal strategies are sparse.
Mixed vaginitis/co-infections	Concurrent BV, VVC, AV, or other infections; often more severe symptoms and higher treatment failure.	Gut may sustain repeated introduction of bacterial and fungal agents; BV biofilm can facilitate Candida persistence.	Polymicrobial biofilms, antibiotic-driven Lactobacillus depletion, local metabolite shifts, and fungal-bacterial interactions reinforce recurrence.	Treat the identified components rather than assuming a single etiology; avoid broad regimens that worsen dysbiosis when possible.	Clinical heterogeneity is high; definitions and diagnostic criteria vary.
Recurrent urogenital infections/UTI-adjacent phenotype	Vaginal dysbiosis may coexist with recurrent UTIs or uropathogen carriage.	Rectal and vaginal reservoirs of uropathogens can seed the urinary tract; vaginal Lactobacillus depletion may increase susceptibility.	Gut-rectal-vaginal-urinary reservoir dynamics can maintain recurrence in susceptible women.	Integrated phenotyping of gut, rectal, vaginal, and urinary reservoirs may improve prevention strategies.	This extends beyond the vagina alone; keep the framing as gut–urogenital when urinary outcomes are central.

**Table 3:** Therapeutic modulation of the gut–vagina axis.

Intervention Layer	Examples/Targets	Axis Target	Potential Use in the Manuscript	Evidence Strength	Key Caveats
Oral probiotics	*Lactobacillus rhamnosus* GR-1 + *Lactobacillus reuteri* RC-14; other oral Lactobacillus combinations.	Gut reservoir, perineal transfer route, systemic immune/metabolic signaling, and possible vaginal colonization.	Discuss as proof-of-concept that oral modulation can influence urogenital outcomes, especially BV adjunct therapy, VVC, UTIs, and recurrence prevention.	Moderate for specific strains/products; not generalizable to all probiotics.	Effects are strain-, dose-, formulation-, timing-, route-, and host-dependent; avoid generic claims such as “probiotics work”.
Intravaginal live biotherapeutics	*Lactobacillus crispatus* CTV-05/Lactin-V after standard BV therapy. *Lactobacillus plantarum* P17630 after standard systemic therapy for vaginal recurrent infections.	Direct restoration of vaginal Lactobacillus dominance and ecological resilience.	Use as the clearest example of targeted vaginal microbiome restoration and recurrence reduction.	Moderate for BV recurrence (*L. crispatus*) and VVC or vaginal infections (*L. plantarum*) in studied settings.	Colonization success varies; product availability, baseline microbiota, menses, sex, and recent antibiotics matter.
Other Lactobacillus candidates	*L. crispatus*, *L. gasseri*, *L. jensenii*, *L. plantarum*, *L. acidophilus*, *L. delbrueckii* and others.	Vaginal eubiosis, lactate production, pathogen exclusion, epithelial adherence.	Mention as candidates, but emphasize that species identity is less important than strain-level function.	Low-moderate depending on strain and indication.	Do not extrapolate from one Lactobacillus strain to another; *L. iners* should be discussed carefully because it may be less protective.
Prebiotics	Lactulose, lactitol, raffinose, oligofructose, inulin, pectin and related substrates.	Selective support of beneficial lactobacilli and possibly gut SCFA-producing taxa.	Position as promising but less clinically proven axis modulation.	Low-moderate; often in vitro or early clinical evidence.	May affect gut and vagina differently; excessive sugar/high glycemic patterns should not be conflated with targeted prebiotics.
Postbiotics	Non-viable microbial products, exopolysaccharides, extracellular vesicles, cell-wall components.	Epithelial adherence, anti-adhesion effects against pathogens, barrier support without live-organism risks.	Useful as future direction for patients where live bacteria are undesirable.	Low/early.	Definitions and products are heterogeneous; clinical vaginal data remain limited.
Synbiotics	Combined probiotic + prebiotic formulations.	Improved survival, engraftment, and functional activity of beneficial strains.	Describe as rational but still product-specific strategy.	Low-moderate.	Synergy must be demonstrated; do not assume every combination is beneficial.
Dietary modulation	Fiber, fruits/vegetables, vitamins A/C/E/D, micronutrients, lower high-glycemic dietary load, moderation of alcohol/animal-protein excess.	Gut microbiome composition, SCFA production, systemic inflammation, estrogen metabolism, vaginal ecological resilience.	Place in a pragmatic prevention section rather than as direct treatment of acute infection.	Low-moderate, mostly observational for vaginal outcomes.	Dietary data are confounded by lifestyle, socioeconomic factors, BMI, medication, and sexual/reproductive variables.
Targeted antimicrobials plus restoration	Condition-specific antibiotics/antifungals followed by Lactobacillus restoration or recurrence-prevention strategy.	Acute pathogen control plus ecological recovery of the vaginal niche and possibly reservoir reduction.	Frame as an integrated strategy for AV, BV, VVC, and mixed infections.	Standard therapies are established; axis-specific integrated evidence is low-moderate.	Broad-spectrum therapy can worsen Lactobacillus depletion or promote Candida; diagnosis must guide treatment.

**Table 4:** Key gaps and research priorities.

Research Priority	Why It Matters	Recommended Study Design	Minimum Measurements	Expected Manuscript Message
Quantify gut -> vagina migration	This is the strongest pillar but still needs frequency, timing, and clinical relevance.	Longitudinal paired rectal/vaginal sampling with strain-resolved metagenomics before, during, and after perturbations.	Species and strain tracking, culture validation, hygiene/sex/antibiotic metadata, pH, CST, symptoms.	Gut-to-vagina seeding is plausible and increasingly supported, but clinically meaningful colonization requires proof of persistence and phenotype change.
Test vagina -> gut directionality	The word “bidirectional” is reviewer-sensitive unless asymmetry is explicit.	Dense multi-site sampling with source-tracking and temporal modeling; include negative controls to reduce contamination concerns.	Vaginal/rectal/gut strains, sampling order, contamination controls, behavioral exposures.	Reverse transfer should be presented as plausible but less established.
Establish estrobolome causality	Hormone regulation is a central and clinically attractive mechanism.	Prospective cohorts or interventions combining fecal enzyme assays, metagenomics, and steroid metabolomics.	Fecal beta-GUS/sulfatase activity, urinary/serum estrogen metabolites, vaginal glycogen, Lactobacillus abundance, pH.	Gut microbial estrogen metabolism may indirectly regulate vaginal ecology, but effect size in humans must be quantified.
Resolve metabolite pathways	Metabolites are biologically plausible but direct vaginal-specific evidence is limited.	Paired fecal/serum/vaginal metabolomics with epithelial and immune readouts.	SCFAs, bile acids, indoles, MAMP markers, tight-junction markers, cytokines, CST, clinical phenotype.	Metabolite exchange should be framed as systemic host-mediated signaling, not bulk luminal transfer.
Map gut–vaginal immune trafficking	Immune homing is conceptually strong but undermeasured in human gut–vagina studies.	Translational studies integrating mucosal immunology, chemokines, antibodies, and microbiome profiles.	IgG/IgA, IgA coating, CCL28/CCR10, T/B cell phenotypes, antimicrobial peptides, cytokines.	Gut immune education may influence vaginal immunity; paired human data are needed.
Define intervention responders	Probiotic and microbiome interventions are heterogeneous and product-specific.	Stratified randomized trials by baseline CST, menopause/pregnancy status, infection type, and recurrence phenotype.	Strain colonization, recurrence rate, symptom resolution, safety, baseline microbiota, antimicrobial exposure.	The field should move from generic probiotics to strain-specific, phenotype-specific live biotherapeutic strategies.
Create an integrated clinical phenotype	Recurrent infections often involve gut, vaginal, urinary, hormonal, and behavioral factors.	Prospective clinical registry with microbiome, metabolome, immune, hormonal, and treatment-response data.	AV/BV/VVC/mixed diagnosis, CST, gut symptoms, urinary symptoms, hormones, diet, antibiotics, recurrence.	The axis is most useful clinically when it organizes recurrent and mixed disease rather than replacing standard diagnoses.

## Data Availability

No new data were created or analyzed in this study. Data sharing is not applicable.

## Abbreviations

AhR	aryl hydrocarbon receptor
AMPs	antimicrobial peptides
AV	aerobic vaginitis
BSH	bile salt hydrolase
BV	bacterial vaginosis
CSTs	community state types
FXR	farnesoid X receptor
GALT	gut-associated lymphoid tissue
GLP-1	Glucagon-like peptide-1
GPCRs	G-protein coupled receptors
GUS	β-glucuronidase
HDACs	histone deacetylases
IBS	irritable bowel syndrome
Ig	immunoglobulin
MAMPs	microbe-associated molecular patterns
NGS	next-generation sequencing
PCOS	polycystic ovary syndrome
SCFAs	short-chain fatty acids
STIs	sexually transmitted infections
SULTs	sulfotransferases
TGR5	Takeda G protein-coupled receptor 5
UGT	UDP-glucuronosyltransferase
UTIs	urinary tract infections
VVC	vulvovaginal candidiasis
